# Efficacy of peritoneal dialysis in patients with refractory congestive heart failure: a systematic review and meta-analysis

**DOI:** 10.1007/s10741-023-10297-3

**Published:** 2023-02-04

**Authors:** Ana Teresa Timóteo, Tânia Branco Mano

**Affiliations:** 1 Cardiology Department, Santa Marta Hospital, Centro Hospitalar Universitário Lisboa Central, Rua Santa Marta, 1169-025 Lisbon, Portugal; 2 NOVA Medical School, Lisbon, Portugal

**Keywords:** Refractory, Congestive heart failure, Peritoneal dialysis

## Abstract

Refractory congestive heart failure (RCHF) is a common complication in the natural history of advanced heart failure. Peritoneal dialysis (PD) is a possible alternative in those patients, but studies are scarce, and mostly with small samples. We conducted this meta-analysis to evaluate the effects of PD in patients with RCHF. Articles published before July 2020 in the following databases: PubMed, Web of Science, and CENTRAL. Mean differences (MD) and 95% confidence intervals (CIs) were computed to generate a pooled effect size with a random effects model. We also assessed heterogeneity, risk of bias, publication bias, and quality of evidence. Twenty observational studies (*n* = 769) were included, with a “before and after intervention” design. PD was associated with a significant reduction in NYHA functional class (MD −1.37, 95% CI −0.78 to −1.96) and length of hospitalisation (MD −34.8, 95% CI −20.6 to −48.9 days/patient/year), a small but significant increase in left ventricular ejection fraction (MD 4.3, 95%CI 1.9 to 6.8%) and a non-significant change in glomerular filtration rate (MD −3.0, 95% CI −6.0 to 0 mL/min/1.73m2). Heterogeneity among studies was significant and overall risk of bias was rated from moderate to critical. No significant publication bias was found, and the overall quality of evidence was very low for all outcomes. PD in patients with RCHF improved functional class, length of hospitalisation, and ventricular functional, and had no impact in renal function. Further randomised clinical trials are warranted to confirm our results that showed some limitations.

**Supplementary Information:**

The online version contains supplementary material available at 10.1007/s10741-023-10297-3

## Introduction

Cardiovascular diseases are the leading cause of mortality in developed countries, including Europe [1]. Heart failure is a terminal stage in the natural history of patients with cardiovascular diseases. In the last decades, significant progress has been made in the treatment of heart failure, particularly in heart failure with reduced ejection fraction, with several disease-modifying drugs and increasingly complex devices [2, 3]. However, in some patients, the effectiveness of therapy is limited, and the only available option is palliative care to achieve some late improvement in quality of life.

In heart failure patients, congestion is a very important limiting factor for the quality of life and in very advanced stages, its control can be difficult, especially with the development of diuretic resistance, which can occur in up to 50% of hospitalised patients with acute congestive heart failure [4]. This resistance is multifactorial, and it can be related to impaired renal function, disrupted pharmacokinetics of diuretics, intravascular fluid depletion, reduced renal perfusion, activation of the renin–angiotensin–aldosterone and sympathetic systems, and compensatory distal tubular reabsorption of sodium [5].

Improvements in heart failure treatment increased survival, and refractory congestive heart failure (RCHF) is a growing health problem, being already an important cause of hospitalisation, with the associated costs [6, 7]. With diuretics resistance, extracorporeal haemodialysis or ultrafiltration is an alternative to treat congestion. However, it does not relieve the burden on hospital services, because it must be performed in a hospital setting, and clinical studies, such as the UNLOAD and CARRESS HF trial, yielded conflicting results [8, 9]. Peritoneal ultrafiltration with or without dialysis (PD) is also another alternative, with the advantage of being continuous and slow, allowing the removal of the extracellular fluid in a more physiological way, without interfering with the patient’s hemodynamic stability [4, 5]. In selected cases, it can be performed on an outpatient/home-based setting, with lower costs. Existing studies in the literature are mostly small and observational and therefore, there is lack of solid evidence on its use in heart failure. Previous systematic reviews found that hospitalisation days declined significantly, with improvements in New York Heart Association (NYHA) class and Left Ventricular Ejection Fraction (LVEF) [10, 11]. However, more recent studies were not included. For that reason, our objective is to summarise and analyse data reported in the literature, to obtain more up-to-date and consistent data on the effectiveness of PD in patients with refractory congestive heart failure.

## Methods

This study was performed and reported according to the Preferred Reporting Items for Systematic Reviews and Meta-Analysis (PRISMA) statement [12]. All stages of study selection, data extraction, and quality assessment were performed independently by two reviewers. Any disagreement was resolved through discussion and consensus.

### Literature search

We performed electronic database search in PubMed, Web of Science Core Collection, and Cochrane Central Register of Controlled Trials (CENTRAL) from database inception until July 2020, for articles meeting our inclusion criteria. A combination of Medical Subject Headings and text words using Boolean search strategies was used to identify studies. The following terms adapted to each database and in various combinations were used for the search: “heart failure”, “cardiac failure”, “ventricular dysfunction”, “peritoneal dialysis”, and “peritoneal ultrafiltration”. Our search did not have any language or geographical restrictions. In addition, relevant reviews obtained in the searching process as well as the references of included studies were manually analysed to search for potential additional eligible studies that were not identified in the database computer search.

### Study selection

All titles and abstracts retrieved by the search were reviewed independently by two authors to identify potentially relevant articles for full-text review. Selected studies underwent full-text assessment to determine the appropriateness for inclusion.

### Eligibility criteria

Inclusion and exclusion criteria were set before data extraction.

Inclusion criteria were as follows: (1) prospective or retrospective design; (2) observational cohort or randomised clinical trial design; (3) adult population (age ≥ 18 years); (4) diagnosis of refractory congestive heart failure, as defined by the 2016 ESC Guidelines for the diagnosis and treatment of acute and chronic heart failure; (5) at least five patients treated with peritoneal dialysis; (6) pre and post-studies or comparative studies with other treatment strategies; and (7) report of at least two of the study outcomes at 6 to 12 months after initiation of PD treatment.

Exclusion criteria were as follows: reviews, editorials, letters to the editor, case reports, conference abstracts, unpublished studies, and animal experimental studies. For multiple publications from the same cohort, we chose the latest or most complete study for assessment. Studies in patients treated with PD before 1995 were also excluded.

Because this is a meta-analysis of previously published articles, ethics committee approval, and informed consent is waived.

### Data extraction

Data from each study was extracted with standardised forms: first author; year of publication; country; period of enrolment; study design; mean follow-up; number of patients in each study; demographic; and study population features.

The following clinical outcomes were used to assess the efficacy of PD therapy: (1) hospitalisation duration; (2) heart function by LVEF; (3) NYHA functional classification; and (4) renal function by estimated glomerular filtration rate (GFR); we also analysed adverse clinical outcomes: peritonitis rate and all-cause mortality. The mortality rate was assessed at 1-year follow-up. Peritonitis was reported as the number of episodes per patient/year. All other outcomes were analysed as the difference before and after PD treatment.

### Quality assessment

The risk of bias was independently evaluated by two authors using the Risk of Bias in Non-randomised Studies–of Interventions (ROBINS-I) tool, assessing the following domains: confounding, selection of participants, classification of intervention, deviations from the intervention, missing data, measurement of outcome, and selection of reported results [13]. These domains were qualitatively classified as at critical, serious, moderate, or low risk of bias. The overall risk of bias for each study was divided following ROBINS-I criteria. Risk of bias graphs were derived from this tool [14].

We used the Grading of Recommendations, Assessment, and Evaluation (GRADE) framework to report the overall quality and strength of the evidence per outcome [15]. The certainty in the evidence for each outcome was graded as high, moderate, low, or very low. Tables were prepared with GRADEpro™.

### Statistical analysis

Statistical analyses were performed using Review Manager 5.4.1™ software. A few studies report continuous data as median and interquartile range. We used Wan and Luo formulas for imputing a missing mean and standard deviation value based on the lower quartile, median, and upper quartile summary statistics [16, 17]. They assume normally distributed outcomes but have been observed to perform well when analysing skewed outcomes [18]. A summary statistic was calculated for each study to describe the observed intervention effect. We used by default the inverse variance statistical method and the random-effects model (irrespective of the heterogeneity) to estimate pooled data. The effect measure is reported as mean difference (MD) and 95% Confidence Intervals (CI). MD represents the absolute difference between “before” and “after” intervention outcomes. Individual studies and meta-analysis estimates were derived and presented in forest plots.

Heterogeneity among studies was measured through the Cochrane’s *Q* test to calculate the *I*^2^ statistic that estimates the percentage of total variation between studies[19]. Based on *I*^2^, heterogeneity was rated as low (*I*^2^ < 50%), moderate (50–75%), or high (> 75%). When analysis revealed high heterogeneity, we further conducted sensitivity analysis by excluding one study at a time to reflect the effect of the specific data on the overall effect size and the stability of the results. Sensitivity analyses were also performed, by excluding studies at critical risk of bias and further excluding studies at serious and critical risk of bias.

Publication bias was assessed through visual inspection of asymmetry in funnel plots and quantitatively analysed by the Begg and Mazumdar’s rank correlation test, and the Egger’s linear regression test [20, 21]. Publication bias and publication year report were assessed with ProMeta3™ software.

A *p*-value < 0.05 was considered to indicate statistical significance.

## Results

### Included studies

**Fig. 1:**
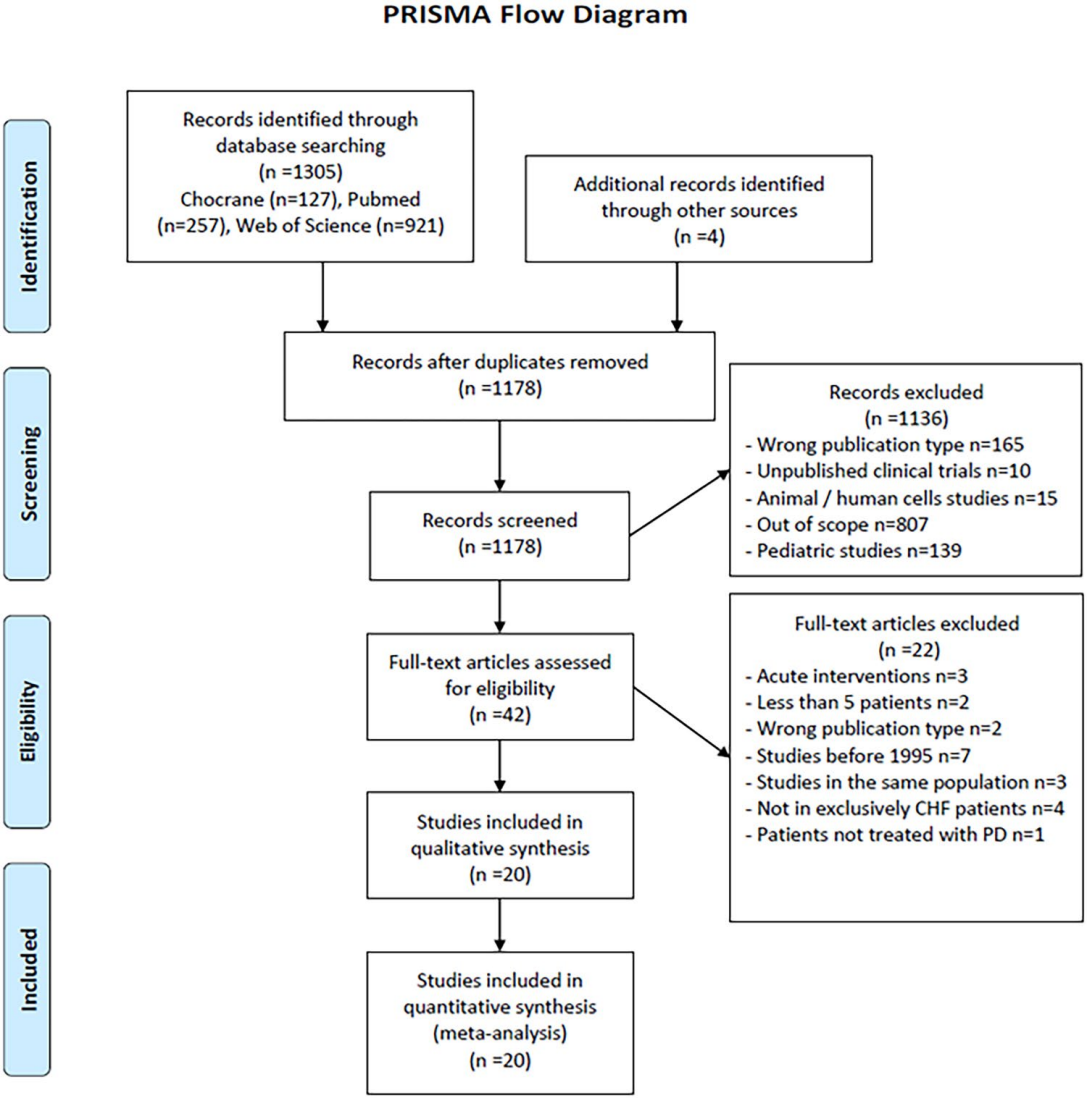
Flow chart of article selection (PRISMA flow diagram). CHF, congestive heart failure; PD, peritoneal ultrafiltration/dialysis

The search returned 1309 records, resulting in 1178 studies after removing 131 duplicates. After title and abstract screening, 43 articles underwent full-text screening, with 20 being included for qualitative and quantitative analysis (Fig. 1) [22–41]. There were no randomised controlled trials, and all studies have a pre- and post-intervention design. The main characteristics of the included studies are detailed in Table 1. Overall, there were 769 patients involved, mostly males, with mean age ranging from 54 to 81 years. Patients were treated from 1995 to 2017 and study’s country of origin is mainly from Europe, Middle East, and Asia. A total of 12 studies had a prospective design and all the others were retrospective. Mean follow-up ranged from 9 to 29 months.

**Table 1:** Characteristics of studies included in the systematic review

Authors	Year publication	Region	Years of enrolment	Study design	Mean follow-up (months)	Patients (n)	Age (years)	Males (%)	Population
Grosskettler et al. [22]	2019	Germany	Jan 2010–Dec 2014	Prospective, observational, multicentre registry	13.3	159	73 ± 12	83.7	Refractory, end-stage CHF with reduced ejection fraction, on OMT, with at least 2 cardiac hospitalisations in the last 6 months, and contraindication for heart transplant
Wojtaszek et al. [23]	2019	Poland	Jan 2005–Dec 2017	Prospective, single centre	24.0	15	72 ± 9	87.0	Refractory CHF, on OMT, with at least 3 cardiac hospitalisations in the last 12 months, and contraindication for heart transplant
Shao et al. [24]	2018	China	Jan 2007–Dec 2010	Prospective, single centre	13.1	14	54 ± 15	57.1	Refractory CHF on OMT, with at least 2 cardiac hospitalisations in the last 12 months
Pavo et al. [25]	2018	Austria	Jan 2009–Jul 2016	Prospective, cohort study, single centre	13.3	40	n.a	n.a	Refractory right ventricular dysfunction, on OMT, and at least 2 hospitalisations in the last 6 months, with CRS type 2
Hedau et al. [26]	2018	India	n.a	Prospective, open-label, single centre	6	30	63 ± 7	70.0	Refractory CHF despite maximal OMT, with at least 2 hospitalisations in the past 6 months
Querido et al. [27]	2016	Portugal	Dec 2008–Jan 2012	Retrospective, observational, single centre	9.4	5	62 ± 16	60.0	Refractory CHF, despite OMT and contraindication for heart transplant
Frohlich et al. [28]	2015	Germany	2006–2012	Prospective, single centre	9.5	39	n.a	n.a	Refractory, end-stage CHF on OMT, and at least 2 hospitalisations in the past 6 months, not suitable for heart transplant
Bertoli et al. [29]	2014	Italy	Jan 2006–Dec 2010	Retrospective, multicentre	24.0	48	74 ± 9	81.3	Refractory CHF on OMT
Courivaud et al. [30]	2014	France	Jan 1995–Dec 2010	Retrospective, multicentre	NA	126	72 ± 11	69.0	Refractory CHF, not candidate for heart transplant
Ritzkallah et al. [31]	2013	Canada	2007–Mar 2011	Retrospective, single centre	NA	10	58 ± 13	70.0	Refractory, end-stage CHF on OMT, diuretic resistance, recurrent hospitalisations, transplant-ineligible
Kunin et al. [32]	2013	Israel	Jul 2008–Dec 2011	Prospective, observational, single centre	14.0	37	66 ± n.a	73.0	Refactory CHF on OMT
Nunez et al. [33]	2012	Spain	Aug 2008–Feb 2011	Prospective, cohort, observational, single centre	16.0	25	75 ± 8	72.0	Refractory CHF despite OMT, and at least 2 non-planned admissions for acute heart failure in the last 6 months, and GFR < 60
Ruhi et al. [34]	2012	Turkey	n.a	Retrospective, single centre	6	6	n.a	83.3	Refractory CHF on OMT and frequent hospitalisations for acute heart failure
Koch et al. [35]	2012	Germany	Mar 2002–Mar 2011	Prospective, Non-randomised, observational, single centre	13.3	118	73 ± 11	60.2	Advanced refractory CHF with impaired renal function
Sotirakopoulos et al. [36]	2011	Greece	Jan 1999–Jan 2007	Retrospective, single centre	NA	19	71 ± 8	n.a	Severe CHF, with frequent hospitalisations for AHF, resistant to diuretics, and without end-stage renal disease
Sanchez et al. [37]	2010	Spain	Dec 2004–Nov 2008	Prospective, single centre	15.0	17	64 ± 9	64.7	Refractory CHF complicated by severe renal failure (CRS type 2)
Cnossen et al. [38]	2010	The Netherlands	Aug 1997–Jan 2008	Retrospective, single centre	12.1	24	67 ± 10	75.0	Age ≥ 70 years, stage 3–5 CKD, at least 3 hospitalisations in the last 12 months, despite OMT (refractory CHF)
Nakayama et al. [39]	2010	Japan	Apr 2002–May 2008	Prospective, single centre	29.4	12	81 ± 6	58.3	Severe refractory CHF on OMT, reduced EF, ineligible for heart transplant
Diaz-Ojea et al. [40]	2007	Spain	Dec 2004–May 2007	Retrospective, single centre	9.8	5	60 ± 6	40.0	Severe refractory CHF on OMT
Gotloib et al. [41]	2005	Israel	2000–2003	Prospective, non-randomised, single centre	19.8	20	66 ± 8	n.a	Severe refractory CHF on OMT

*CHF* congestive heart failure, *GFR* estimated glomerular filtration rate, *CKD* chronic kidney disease, *CRS* cardio-renal syndrome, *n.a.*, not available, *OMT* optimised medical therapy

### Quality of evidence evaluation

The overall risk of bias of the included studies was rated from moderate to critical (Supplemental Figs. 1 and 2) and 60% of the studies had serious or critical risk of bias. The main reason for this classification was bias due to missing data because most studies do not report data after intervention from all subjects included at baseline—mortality rate is high, and some patients were lost to follow-up. Another important cause of bias was some deviations from the intended intervention, such as the transition to haemodialysis due to failure or complications related to PD.

**Fig. 2:**
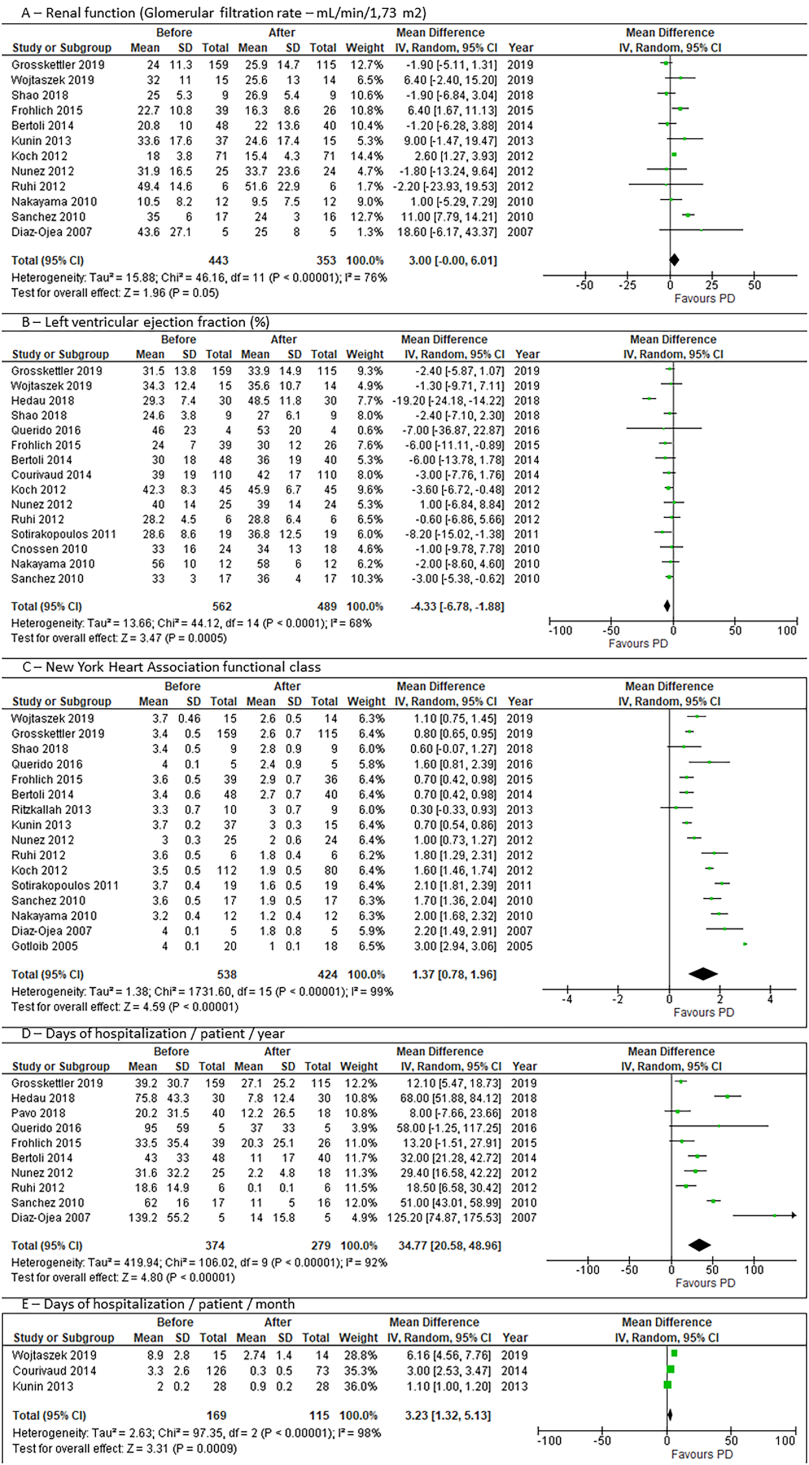
Forest plots of the pooled analysis for each main outcome. CI, confidence interval; IV, inverse variance; PD, peritoneal ultrafiltration/dialysis; SD, standard deviation

No publication bias was found in most of the analysed outcomes with Begg and Mazumdar test. Funnel plots are depicted in Supplementary Fig. 3.

The GRADE confidence for all main outcomes estimates is very low (Supplemental Fig. 4).

### Renal function

A total of 12 observational studies (*n* = 4 43) contributed with data for this outcome. At baseline, mean GFR ranged from 10.5 to 49.4 mL/min/1.73 m^2^. Pooled results showed a very small and non-significant decrease of GFR after PD initiation (MD −3.0 mL/min/1.73 m^2^, 95% CI −6.0 to 0, *p* = 0.05) (Fig. 2). Moderate statistical heterogeneity (*I*^2^ = 76%, *p* < 0.0001) was present for the overall pooled results. Sensitivity analysis showed that after the exclusion of the individual studies by Grosskettler, Shao, Bertoli, Nunez, and Ruhi, mean differences and 95% CI became significant, ranging from −3.1 to −3.7 mL/min/1.73 m2, all with a significant *p*-value. Sensitivity analysis with the removal of the critical risk of bias studies, showed a pooled effect that remained non-significant (MD −3.6, 95% CI −7.7 to 0.59, *p* = 0.09, *I*^2^ = 77%), as well as after removal of the serious and critical risk of bias studies (MD −0.9, 95% CI −5.9 to 4.1, *p* = 0.72, *I*^2^ = 18%). No publication bias was found with Egger’s test (*p* = 0.854) or Begg and Mazumdar’s test (*p* = 0.411).

### Left ventricular ejection fraction

Fifteen studies (*n* = 562) reported LVEF before and after the intervention, with means in the range between 24 and 56% before intervention. Pooled analysis showed that after PD initiation, there was a statistically significant increase in LVEF (MD 4.33%, 95% CI 1.88 to 6.78%, *p* < 0.0001) (Fig. 2). There was also moderate heterogeneity (*I*^2^ = 68%, *p* < 0.001). Sensitivity analysis showed consistent significant differences in effect after the intervention, with increases ranging from 3.2 to 4.7%. In additional sensitivity analysis with the removal of the critical risk of bias studies, the pooled effect remained significant (MD 4.3%, 95% CI 0.81 to 7.83%, *p* = 0.02, *I*^2^ = 79%), but not after removal of the serious and critical risk of bias studies (MD 5.03%, 95% CI −0.12 to 10.18%, *p* = 0.06, *I*^2^ = 85%). No publication bias was found with Egger’s test (*p* = 0.764) or Begg and Mazumdar’s test (*p* = 0.656).

### New York Heart Association (NYHA) functional class

Sixteen studies (*n* = 538) reported the change in NYHA class and pooled results showed a significant improvement after intervention (MD −1.37, 95% CI −0.78 to −1.96, *p* < 0.0001), but with very high heterogeneity (*I*^2^ = 99%, *p* < 0.0001) (Fig. 2). Sensitivity analysis showed persistent significant differences in effect, with reductions in NYHA functional class ranging from 1.25 to 1.44. In additional sensitivity analysis with the removal of the critical risk of bias studies, the pooled effect remained significant (MD −1.60, 95% CI −1.00 to −2.19, *p* < 0.00001, *I*^2^ = 99%), as well as after removal of the serious and critical risk of bias studies (MD −1.31, 95% CI −0.81 to −1.81, *p* < 0.0001, *I*^2^ = 90%). Significant publication bias was found with Egger’s test (*p* = 0.012) but not with Begg and Mazumdar’s test (*p* = 0.126). Publication year had a significant impact on effect size, with smaller improvements in NYHA in more recent studies (Supplemental Fig. 5).

### Length of hospitalisation

A total of 10 observational studies (*n* = 374) reported the length of hospitalisation as days of hospitalisation/patient/year, with means ranging from 31.6 to 139.2. Pooled results showed a significant decrease after PD initiation (MD −34.8 days/patient/year, 95% CI −20.6 to 48.9, *p* < 0.0001) (Fig. 2). High statistical heterogeneity (*I*^2^ = 92%, *p* < 0.0001) was present for the overall pooled results. No individual study had a substantial impact on the pooled effect size, ranging from –30.06 to −38.08 days. In addition, sensitivity analysis with the removal of the critical risk of bias studies showed that the pooled effect remained significant (MD 49.9, 95% CI 29.1 to 70.7, *p* < 0.00001, *I*^2^ = 91%), as well as after the removal of the serious and critical risk of bias studies (MD 52.1, 95% CI 22.7 to 81.6, *p* = 0.0005, *I*^2^ = 92%). The funnel plot demonstrated slight asymmetry, suggesting a possible publication bias. However, neither Egger’s test (*p* = 0.348) nor Begg’s test (*p* = 0.655) revealed evidence of publication bias. Three studies (*n* = 169) reported results as days of hospitalisation/patient/month and were analysed separately, also showing a significant reduction of 3 days (Fig. 2).

### Adverse clinical outcomes at 1 year

All-cause mortality at 1 year is reported in 17 studies and a mean value of 37.6% was obtained (Table 2). The other studies did not report mortality or only considered for the study patients that survived at least 12 months. Incidence of peritonitis, one of the most common complications of PD, is reported in 10 studies, and it ranged from 0 to 0.75 episodes/patient/year (Table 2).

**Table 2:** Adverse outcomes (mortality and peritonitis rates)

Author	Mortality at 12 months (%)	Peritonitis (episodes/patient/year)
Grosskettler et al. [22]	39.6	n.a
Wojtaszek et al. [23]	6.6	0.17
Shao et al. [24]	9.0	0.09
Pavo et al. [25]	45.0	0.31
Hedau et al. [26]	n.a	n.a
Querido et al. [27]	60.0	n.a
Frohlich et al. [28]	n.a	n.a
Bertoli et al. [29]	14.6	0.27
Courivaud et al. [30]	42.0	0.46
Ritzkallah et al. [31]	50.0	n.a
Kunin et al. [32]	59.5	0.32
Nunez et al. [33]	28.0	0.75
Ruhi et al. [34]	0*	0
Koch et al. [35]	45.0	0.05
Sotirakopoulos et al. [36]	31.6	n.a
Sanchez et al. [37]	18.0	0.02
Cnossen et al. [38]	50.0	n.a
Nakayama et al. [39]	0	n.a
Diaz-Ojea et al. [40]	20.0	n.a
Gotloib et al. [41]	10.0	0.27

^*^At 6 months. *n.a.*, not available

## Discussion

In this updated meta-analysis on the efficacy of PD in adult patients with RCHF, we retrieved 20 studies, representing a total of 769 patients. All were observational and non-randomised. When measured by the NYHA functional class, almost all studies showed that PD improved symptoms. There was also a positive effect on LVEF with improvements in the range between 1 and 19%. Another important benefit was a significant decline in hospitalisation days by almost 35 days/patient/year. Renal function remained stable during PD treatment, suggesting that it can avoid or delay further deterioration in renal function.

With effective control of volume overload and congestion, it is possible to reduce hospitalisations due to congestion, which we confirmed in our meta-analysis. This reduction can be considered an indirect marker of improved quality of life in these patients and a surrogate marker of better control of heart failure symptoms. We also confirmed a reduction in NYHA functional class. Moreover, as Sanchez demonstrated, total healthcare costs associated with PD were lower when compared to conservative therapy [37]. PD is also associated with a higher utility than the conservative therapy. Cost-utility for PD was, at that time, 23 305€/quality-adjusted life-year (QALY), while for conservative treatment it was 81 053€/QALY, with a difference of 46 237€ per QALY. PD is cost-effective compared with the conservative therapy and this is very important when the economic burden of heart failure is expected to increase in the next years.

We observed a slight improvement in left ventricular function. Effective decongestion by PD decreases preload that can theoretically improve ejection fraction, not only by allowing a reduction in the activation of both renin–angiotensin–aldosterone axis and sympathetic nervous system but also by another possible contributing factor related to the removal of myocardial depressant factors [26].

Renal function remained stable after PD in patients without end-stage chronic kidney disease. This may be related to improvement in renal perfusion, secondary to improved cardiac function and reduced neurohormonal activation [10, 26]. This can also be related to a reduction in renal venous congestion, with general improvement in renal hemodynamics [10, 26].

Patients with refractory congestive heart failure have a very ominous prognosis, not only in the quality of life but also in survival. Our population of patients had multiple previous hospitalisations for congestive heart failure. Previous studies showed a direct increase in all-cause mortality with the increase in the number of hospitalisations for heart failure. In a patient database of almost 15,000 patients hospitalised for heart failure between 2000 and 2004, 1-year mortality was 34% after the first hospitalisation, reaching 50% after the third hospitalisation [42]. More recent data (2007–2011), showed some improvement, being 27% at 1-year after first hospitalisation and 40% after the third hospitalisation [43]. Similar data is reported in another study with all-cause mortality at 1 year of 36.8–45.2% in patients with recurrent hospitalisations for acute decompensated heart failure, particularly in patients with heart failure with reduced ejection fraction [44]. Our meta-analysis reported a pooled mortality rate slightly lower, of 37.2%, when compared to this historical mortality rate, suggesting that this strategy possibly does not have a very significant impact on survival.

The most common complication of PD is peritonitis, and our results seem to be in line with those reported for chronic PD in end-stage kidney disease patients. In the general population of patients submitted to PD, peritonitis rates in recent publications are reported between 0.26 and 0.37 episodes/patient/year, depending on the technique used—higher for continuous ambulatory peritoneal dialysis [45–47]. Our results have a wide range of incidence, from 0 to 0.75 episodes/patient/year, but most are below 0.32 episodes/patient/year, particularly for studies after 2014, suggesting that this technique is currently safe (regarding infection) in patients with refractory congestive heart failure.

As in the previous meta-analysis, there are important limitations. The overall quality of most studies is poor. They were all observational; length of follow-up was also highly variable; all studies had a pre- and post-intervention design and outcomes of patients that died or were lost to follow-up for any other reason, were not reported. Implications of missing outcome data from those participants are expected to be significant, mainly because they were probably the sickest ones, and a direct comparison between pre- and post-intervention data is not advisable. Missing values were one of the main reasons for the high risk of bias given for most studies. However, analysing only the study outcomes reported in the studies where it was possible to extract specific information from the subset of patients who report both baseline and post-intervention measurements, the null effect in glomerular filtration rate is consistent, as well as the positive impact on NYHA functional class and length of hospitalisation and the effect in LVEF is either neutral or positive supporting the validity of our results [24, 26, 27, 30, 34–37, 39, 40].

There was also high heterogeneity of the pooled studies for most outcomes explained by differences regarding sample size and baseline characteristics between studies. A recent study showed that hospitalisation reductions were only significant in patients with heart failure with preserved ejection fraction and significant improvement in LVEF was only observed in patients with heart failure with reduced ejection fraction, showing the impact of heterogeneity [48].

There are other limitations. Some patients received haemodialysis due to failure of PD treatment and this is another cause for increased risk of bias. Most studies do not report appropriately pharmacological treatment or devices used in the treatment of heart failure, and for that reason, we cannot confirm if the observed change in the clinical outcome can be solely attributed to PD treatment. However, there was a consistent improvement in most outcomes which is something we do not expect in patients with such ominous prognosis.

The lack of prospective randomised controlled trials is also relevant. The peritoneal dialysis in patients with severe heart failure (PD-HF) trial, a multicentre randomised controlled trial of intermittent ultrafiltration by PD plus best standard care versus best standard care for the treatment of RCHF and moderate chronic kidney disease (stages 3–4), was initiated in 2016[49]. Over a 2-year inclusion period, only 10 patients were recruited, and the study was terminated due to the inability to recruit an adequate number of participants. The main reasons reported for ineligibility were fluctuating GFR, sub-optimal heart failure treatment, frailty, and patients being too unwell for randomisation (some patients were considered only when they were at end of life), unwillingness to engage in an invasive therapy, and suboptimal coordination between cardiology and renal services. This example shows the difficulties in engaging a randomised controlled trial, and for the time being, only the evidence presented in meta-analysis is available.

In conclusion, peritoneal dialysis/ultrafiltration in patients with refractory congestive heart failure improved functional class, length of hospitalisation, and left ventricular ejection fraction and had no impact in renal function. These favourable results can also have a very positive economic effect, but further studies on this topic are required. Moreover, randomised clinical trials are warranted to compare this intervention with pharmacological therapy or other treatment strategies regarding survival benefits or symptomatic improvement. This is essential to provide more robust evidence on the best therapeutic option in refractory congestive heart failure because there were important limitations in the studies included.

## Supplementary Information

Below is the link to the electronic supplementary material.

Supplementary file1 (TIF 739 KB)

Supplementary file2 (TIFF 20335 KB)

Supplementary file3 (TIF 300 KB)

Supplementary file4 (TIF 343 KB)

Supplementary file5 (TIF 552 KB)

## Data Availability

This is a meta-analysis of published manuscripts and a summary of those studies is available in Tables 1 and 2. No additional data is provided.
