# Supplementary figures and images for: Efficacy of peritoneal dialysis in patients with refractory congestive heart failure: a systematic review and meta-analysis
Source: Heart Fail Rev. 2023 Feb 4;28(5):1053–63. doi: 10.1007/s10741-023-10297-3 (PMC10403434; doi:10.1007/s10741-023-10297-3)

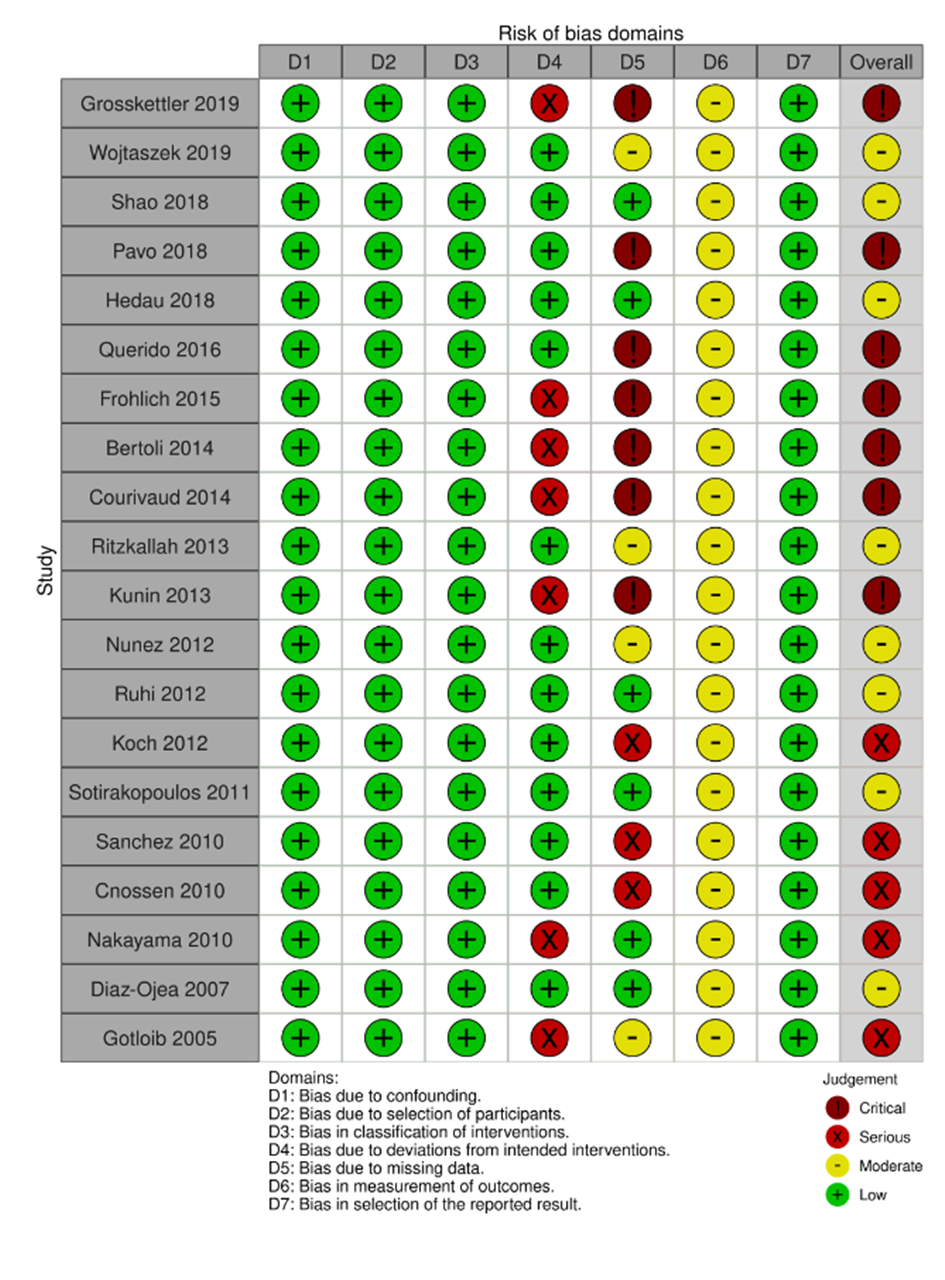

Supplement: Supplementary file 1 — Supplementary file1 (TIF 739 KB) [file 10741_2023_10297_MOESM1_ESM.tif]

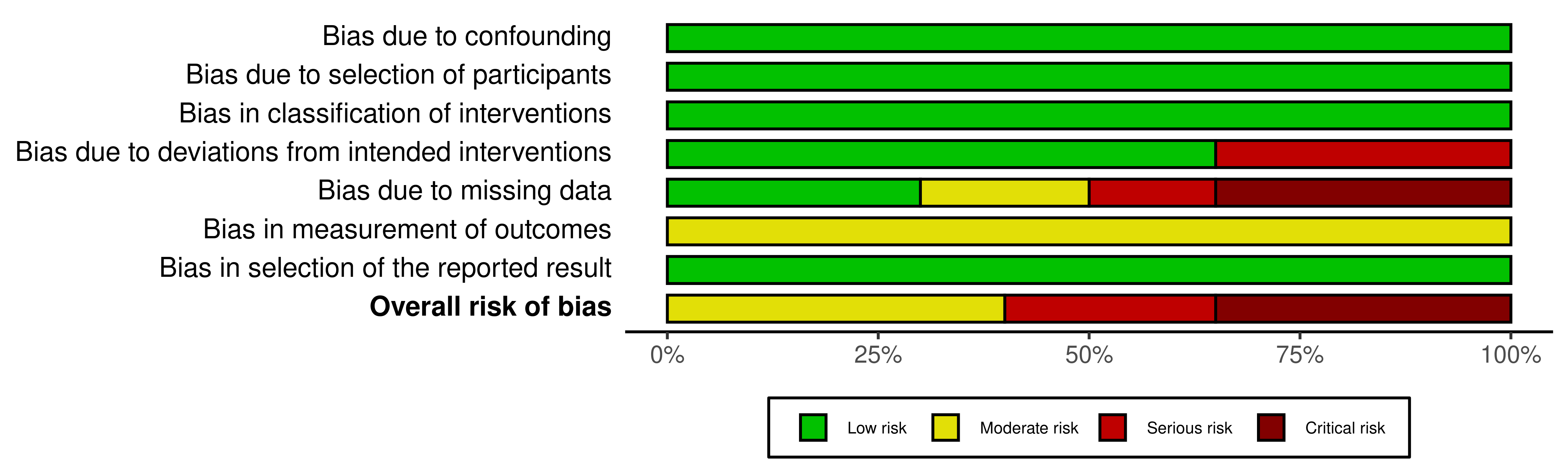

Supplement: Supplementary file 2 — Supplementary file2 (TIFF 20335 KB) [file 10741_2023_10297_MOESM2_ESM.tiff]

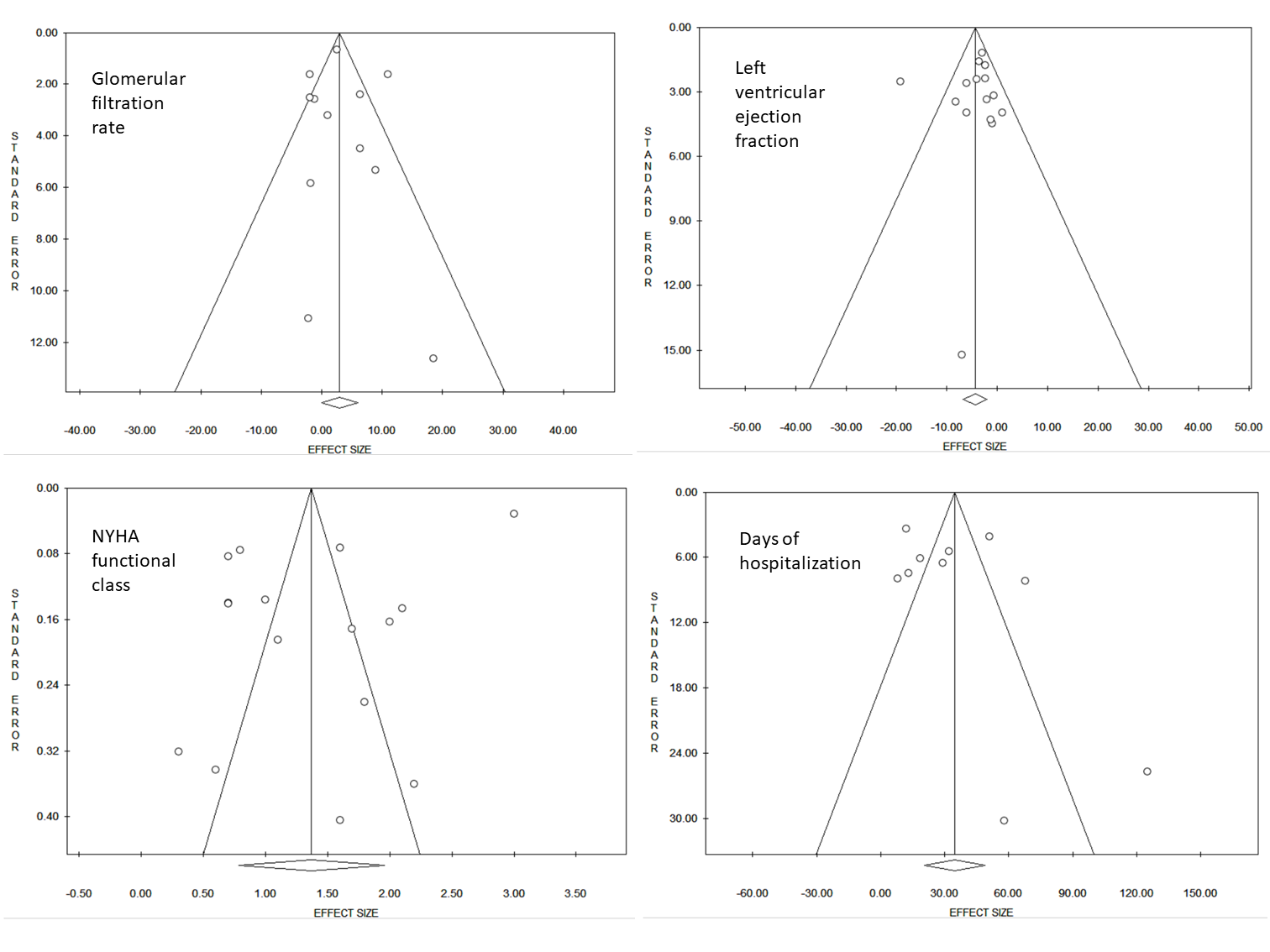

Supplement: Supplementary file 3 — Supplementary file3 (TIF 300 KB) [file 10741_2023_10297_MOESM3_ESM.tif]

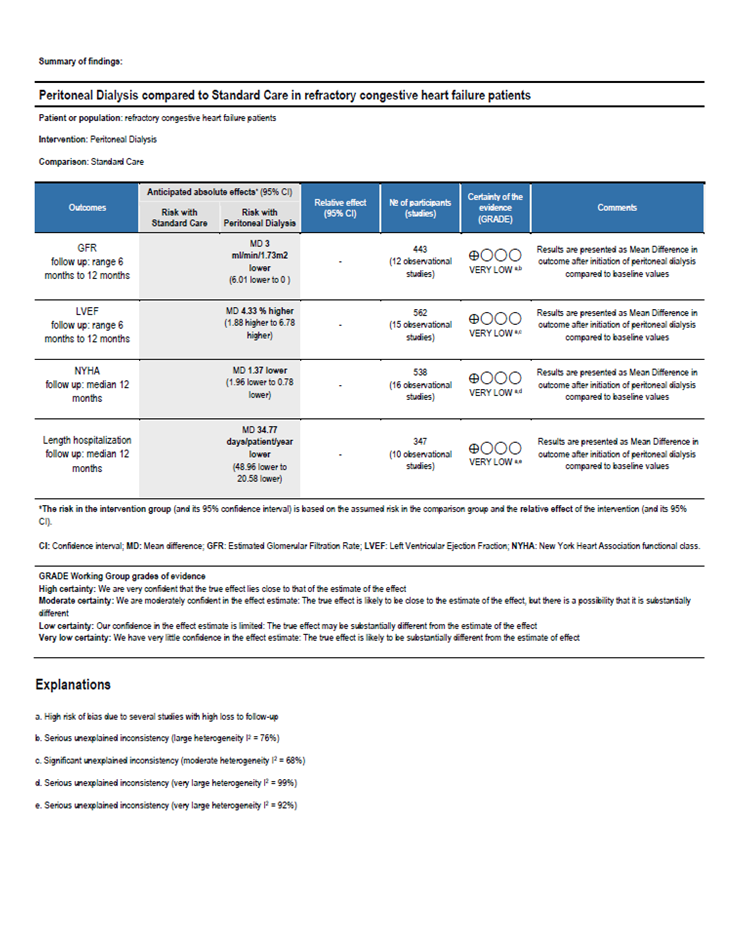

Supplement: Supplementary file 4 — Supplementary file4 (TIF 343 KB) [file 10741_2023_10297_MOESM4_ESM.tif]

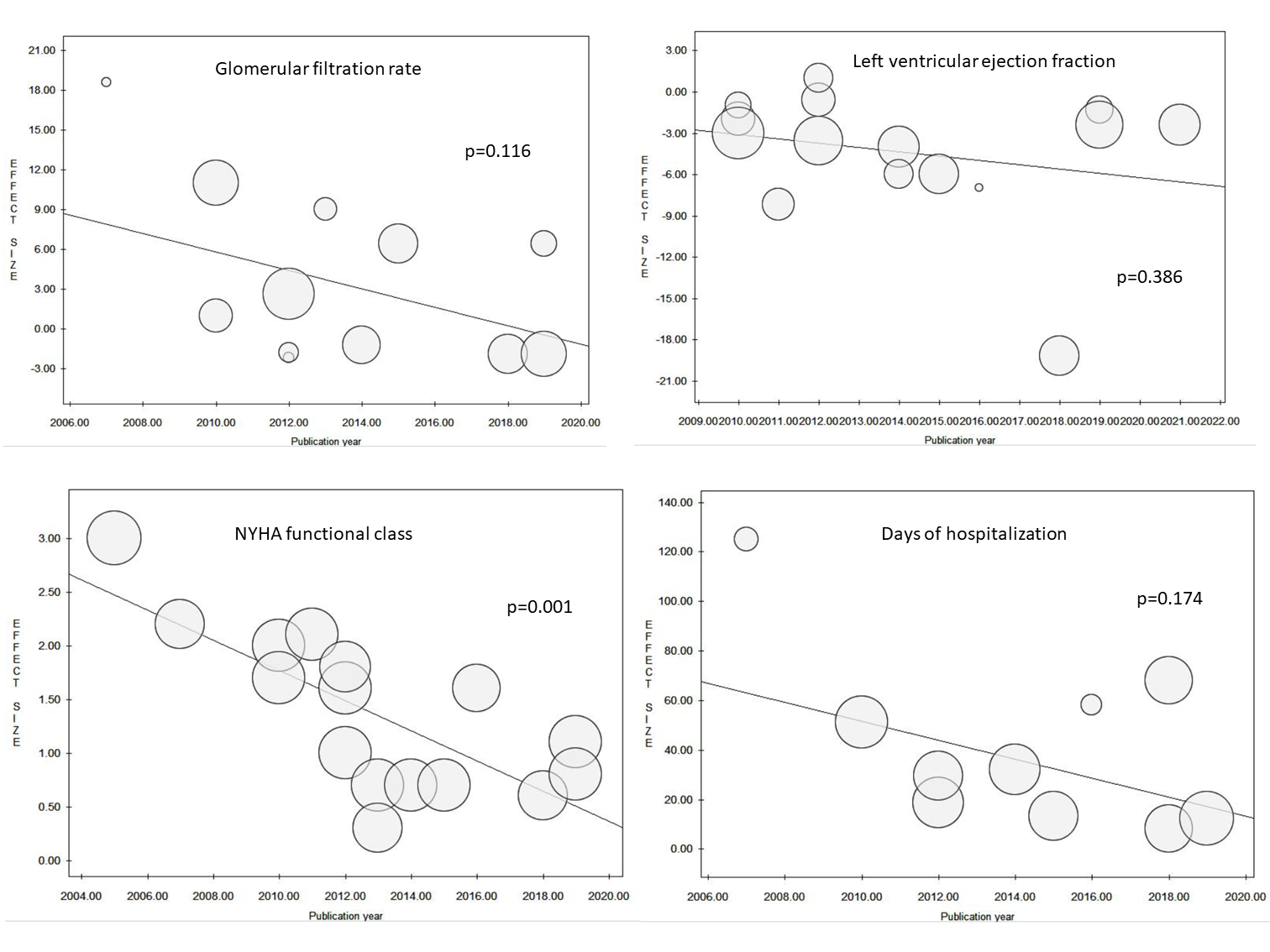

Supplement: Supplementary file 5 — Supplementary file5 (TIF 552 KB) [file 10741_2023_10297_MOESM5_ESM.tif]
